# Effects of walking speed on gait biomechanics in healthy participants: a systematic review and meta-analysis

**DOI:** 10.1186/s13643-019-1063-z

**Published:** 2019-06-27

**Authors:** Claudiane Arakaki Fukuchi, Reginaldo Kisho Fukuchi, Marcos Duarte

**Affiliations:** 1 Neuroscience and Cognition Program, Federal University of ABC, São Bernardo do Campo, São Paulo, Brazil; 2 Biomedical Engineering Program, Federal University of ABC, São Bernardo do Campo, São Paulo, Brazil; 3 Neuroscience and Cognition and Biomedical Engineering Programs, Federal University of ABC, São Bernardo do Campo, Rua Arcturus, 3, São Paulo, SP 09606-070 Brazil; 4 Department of Orthopaedics and Traumatology, Faculty of Medical Sciences, State University of Campinas (UNICAMP), São Paulo, Brazil

**Keywords:** Walking speed, Kinematics, Kinetics, Ground reaction forces, Gait analysis

## Abstract

**Background:**

Understanding the effects of gait speed on biomechanical variables is fundamental for a proper evaluation of alterations in gait, since pathological individuals tend to walk slower than healthy controls. Therefore, the aim of the study was to perform a systematic review of the effects of gait speed on spatiotemporal parameters, joint kinematics, joint kinetics, and ground reaction forces in healthy children, young adults, and older adults.

**Methods:**

A systematic electronic search was performed on PubMed, Embase, and Web of Science databases to identify studies published between 1980 and 2019. A modified Quality Index was applied to assess methodological quality, and effect sizes with 95% confidence intervals were calculated as the standardized mean differences. For the meta-analyses, a fixed or random effect model and the statistical heterogeneity were calculated using the *I*^2^ index.

**Results:**

Twenty original full-length studies were included in the final analyses with a total of 587 healthy individuals evaluated, of which four studies analyzed the gait pattern of 227 children, 16 studies of 310 young adults, and three studies of 59 older adults. In general, gait speed affected the amplitude of spatiotemporal gait parameters, joint kinematics, joint kinetics, and ground reaction forces with a decrease at slow speeds and increase at fast speeds in relation to the comfortable speed. Specifically, moderate-to-large effect sizes were found for each age group and speed: children (slow, − 3.61 to 0.59; fast, − 1.05 to 2.97), young adults (slow, − 3.56 to 4.06; fast, − 4.28 to 4.38), and older adults (slow, − 1.76 to 0.52; fast, − 0.29 to 1.43).

**Conclusions:**

This review identified that speed affected the gait patterns of different populations with respect to the amplitude of spatiotemporal parameters, joint kinematics, joint kinetics, and ground reaction forces. Specifically, most of the values analyzed decreased at slower speeds and increased at faster speeds. Therefore, the effects of speed on gait patterns should also be considered when comparing the gait analysis of pathological individuals with normal or control ones.

**Electronic supplementary material:**

The online version of this article (10.1186/s13643-019-1063-z) contains supplementary material, which is available to authorized users.

## Background

The quantification of the biomechanical characteristics of a person’s gait is an important clinical tool for evaluating normal and pathological patterns of locomotion [1, 2] and has been used in the decision process to prescribe treatment as well as to evaluate the intervention outcomes [3–5]. For example, the walking speed and not age has been considered the primary determinant of the kinematic and kinetic changes in children [6]. In fact, the speed at which a person walks influences biomechanical variables such as joint kinematics, ground reaction forces (GRF), joint moments of force (moments) and powers, muscle activity, and spatiotemporal gait parameters in children [6–9], young adults [10–14], and older adults [15, 16]. However, none of these studies considered all these variables together nor examined different age groups in the same study.

In a typical gait analysis, the gait patterns of pathological individuals are compared with a cohort of healthy individuals walking at their comfortable pace. However, as pathological individuals tend to walk slower and considering different age groups, without knowing which biomechanical variables are likely more affected by gait speed, this comparison may not be appropriate. Thus, to improve the knowledge about the effects of gait speed on biomechanical variables is paramount for benefitting clinicians who commonly rely on the outcomes of gait analysis to optimize patient care [17].

Although there are a handful of studies, including some reviews [18, 19] that examined the influence of walking speed on gait biomechanics, to our knowledge, no study has systematically reviewed the effects of speed on gait over a more comprehensive set of biomechanical variables and across different ages. For example, Telfer and collaborators [18] reported that walking speed has the largest effect on knee abduction moment in individuals over 18 years old, which is related to the development of the medial knee osteoarthritis [20]. Additionally, a systematic review by Herssens and collaborators [19] reported changes in the spatiotemporal parameters in healthy adults between 18 and 98 years old, but only at the self-selected walking speed.

Hence, the aim of the present study was to perform a systematic review of studies that have investigated the effects of gait speed on spatiotemporal parameters, joint kinematics, joint kinetics, and GRF variables in healthy individuals of various ages.

## Methods

### Search strategy

This systematic review was conducted according to the Preferred Reporting Items for Systematic Reviews and Meta-Analyses (PRISMA) statement [21] (Additional file 1) and was registered in PROSPERO (ID122769). All studies were identified by three electronic databases (PubMed, Embase, and Web of Science) which comprise the most topics within the Biomedical and Health Sciences area [22]. The specific search strategy is described in Additional file 2: Table S1.

### Selection criteria

The initial search was completed on December 2017, and on March 2019 a final search using the same terms was performed to verify potential newly published articles. Only original full-length studies published between 1980 and 2019 were included, with the specific inclusion criteria determined a priori: (1) walking as opposed to running; (2) normal (or equivalent), and slow and/or fast speeds measured quantitatively or qualitatively; (3) walking either on a ground or treadmill surface; (4) healthy participants with no orthopedic or neurological disease; (5) gait analysis on a level surface; (6) gait analysis using a three-dimensional (3D) motion capture system or 3D force platforms or both; and (7) article published in English. Reviews, conference papers, abstracts, letters, cases series, and pilot studies were excluded.

Inclusion criteria for the participants were healthy individuals with the age range based on the specific age group: children (4–17 years of age), young adults (18–59 years of age), and older adults (60–85 years of age). Studies that presented individuals with any musculoskeletal or neurological impairment were excluded. Since the aim of this systematic review was not to examine the effect of any intervention, only observational studies (e.g., cohort, case-control, and cross-sectional design) were included in this systematic review.

To ensure identification of all relevant studies, the reference lists of relevant systematic reviews were hand-searched [18, 19, 22].

### Data extraction

All titles returned based on the search terms were first scanned by one of the co-authors, CAF. From the results of the original search, articles were excluded based on the inclusion criteria (e.g., animal study, non-English language, running task, etc.). Following this, all titles and abstracts were reviewed independently by two reviewers, CAF and RKF (co-authors of this article), to determine their eligibility for the study. Whenever there was a disagreement between the two reviewers, the third author was consulted.

Characteristics of studies (authors, year), participants (sample size, age), surface types (treadmill or overground), and gait speed were extracted and reported in Table 1.

**Table 1:** Details of the articles used in the final analysis

Author, year (ref)	Sample size			Age mean (SD)			Surface	Gait speed (m/s)
	Children	Young adults	Older adults	Children	Young adults	Older adults		
de David et al.2015 [23]		11			21.2 (1.8)		Overground	1.61, 2.09
Diop et al. 2005 [24]	94			7.3 (0.6)			Treadmill	0.75, 1.0, 1.25
Dubbeldam et al. 2010 [25]		14			43 (8)		Overground	0.81, 1.28
Giarmatzis et al. 2015 [26]		20			22.2 (1.6)		Treadmill	0.83, 1.25, 1.67
Hsiao et al. 2015 [27]		20			33.5 (20.1)		Treadmill	1.08, 1.30
Kerrigan et al. 1998 [28]		31	31		28.5 (4.9)	72.5 (5.5)	Overground	1.37, 1.19, 1.55
Khan et al. 2017 [29]		20			29 (4.1)		Overground	0.85, 1.18, 1.43
Kwon et al. 2015 [30]		40			23.2 (3.8)		Overground	1.00, 1.50, 2.00
Lewek 2011 [31]		15			27 (9)		Treadmill	0.60, 1.20, 1.60
Linden et al. 2002 [32]	36			9 (0.6)			Overground	0.75, 1.21
Monaco et al. 2009 [33]		9	8		26.4 (2.3)	70.4 (5.3)	Treadmill	0.77, 1.13
Ridge et al. 2016 [34]	14			14.4 (2.1)			Overground	1.23, 1.87
Riley et al. 2001 [35]		24			23.9 (4.4)		Overground	0.87, 1.19, 1.74
Robbins et al. 2009 [36]		32			32 (8)		Overground	1.19, 1.39, 1.60
Schwartz et al. 2008 [7]	83			10.5 (3.5)			Overground	0.65, 1.15, 1.56
Silder et al. 2008 [37]		20	20		26 (3.5)	72.5 (5)	Overground	1.06, 1.33, 1.59
Wang et al. 2017 [38]		15			24.7 (1.2)		Overground	1.1, 1.4, 1.7
Weinhandl et al. 2017 [39]		10			25.8 (6.2)		Overground	1.21, 1.34, 1.48
Winiarski et al. 2019 [40]		20			20.1 (1.2)		Overground	1.04, 1.32, 1.62
Yang et al. 2013 [41]		9			26.4 (2.4)		Treadmill	0.40, 0.93, 1.47

### Methodological quality

All evaluated studies had their quality rated based on a modified version of the Quality Index (QI) tool originally described by Downs and Black [42]. From the original checklist, only item 27 was removed due to its ambiguity [43]. Twenty-six items, comprising the reporting and the external and internal (bias and confounding) validity assessment, were considered in the final analyses, with the maximum score being 27. The following cut-off adopted in this review was based on a previous study that also analyzed the gait kinetics, kinematics, and spatiotemporal parameters but during long-distance running [44]: high quality (≥ 80%), moderate (< 80% and ≥ 47%), and poor quality (< 40%).

### Variables of interest

The following variables were considered in the present study to address the research question: spatiotemporal gait parameters such as step length, stride length, stride time, and cadence; sagittal kinematic and kinetic variables such as hip, knee, and ankle joint angles and joint moments (when available); and horizontal and vertical GRF (for a general description of these variables see [45]). Since knee abduction moment (in the frontal plane) has been reported to be related to the incidence of knee injuries [46, 47], this variable was also analyzed. For consistency, all joint moments are reported as internal ones. For this review, we considered the global maximum and minimum values of the hip, knee, and ankle joint angles in the sagittal plane during the stance and swing phases of the gait cycle. For the joint moments, the maximum and minimum values in the sagittal plane and also the maximum and minimum values of the knee joint in the frontal plane were considered. For the GRF, the first and second peaks of vertical GRF (vertical1 and vertical2, respectively) and the braking and propulsive forces in the anterior-posterior direction were evaluated. Maximum and minimum values of the joint moments and GRF variables were analyzed only during the stance phase. All these variables were included because they have been reported in previous studies within the context of gait analysis [48, 49]. In this review, only studies that provided graphical or numerical data over the gait cycle were considered for further analysis. If a study was initially included in the final list but presented insufficient information, the authors were contacted and asked to provide the data. If they refused, were unable to, or did not respond to the requests, the study was removed from the list.

The effects of gait speed during walking were analyzed separately for children, young adults, and older adults. In cases where the study included sub-groups (i.e., 4–6 years, 6–8 years, 8–10 years), the results of these sub-groups were combined into one group according to the age groups examined in this review (children (4–17 years of age), young adults (18–59 years of age), and older adults (60–85 years of age)). Males and females were also combined. In this review, only the slow, comfortable, and fast speeds were considered for analysis. If any study presented more than three gait speeds (i.e., very slow, slow, comfortable, and fast), the very slow and slow speeds were combined. When the authors did not specify the speed for the comfortable condition, ranges from 1.07 to 1.32 m/s in children [6, 50], 1.05 to 1.43 m/s in young adults [50, 51], and 0.94 to 1.34 m/s in older adults [52] were adopted. Gait speeds below or above the range of each group were considered as slow and fast, respectively.

To account for the effect of gait speed, the effect size (ES) was calculated based on the ratio of the difference between group means of gait speeds and the pooled standard deviation. We compared the comfortable speed with the slow and fast speeds separately where the specific convention was adopted: for the comparison between slow and comfortable speed (slow < comfortable) and for the fast and comfortable speed (fast > comfortable). Additionally, when numerical data were not available but graphs were presented, we manually digitized the graph using the *WebPlotDigitizer* application (https://automeris.io/WebPlotDigitizer/) to obtain the values. The following guidelines were used to interpret the Cohen’s *d* ES [53, 54]: small (0.2–0.5), moderate (0.5–0.8), and large (> 0.8). To calculate the standardized effects across studies, a fixed- or random-effect model was applied based on the following criteria: if the heterogeneity is high (*I*^2^ > 50%), a random-model effect was chosen; contrarily, a fixed effect model was considered [55]. The 95% confidence intervals (CIs) were calculated to evaluate the heterogeneity of the standardized effects. The results for all variables are summarized as effect sizes, lower and upper CIs, standard errors (SEs), Cochran’s heterogeneity statistic (Q), *I*^2^ statistic, and *p* values for the children and older adult groups.

## Results

**Fig. 1:**
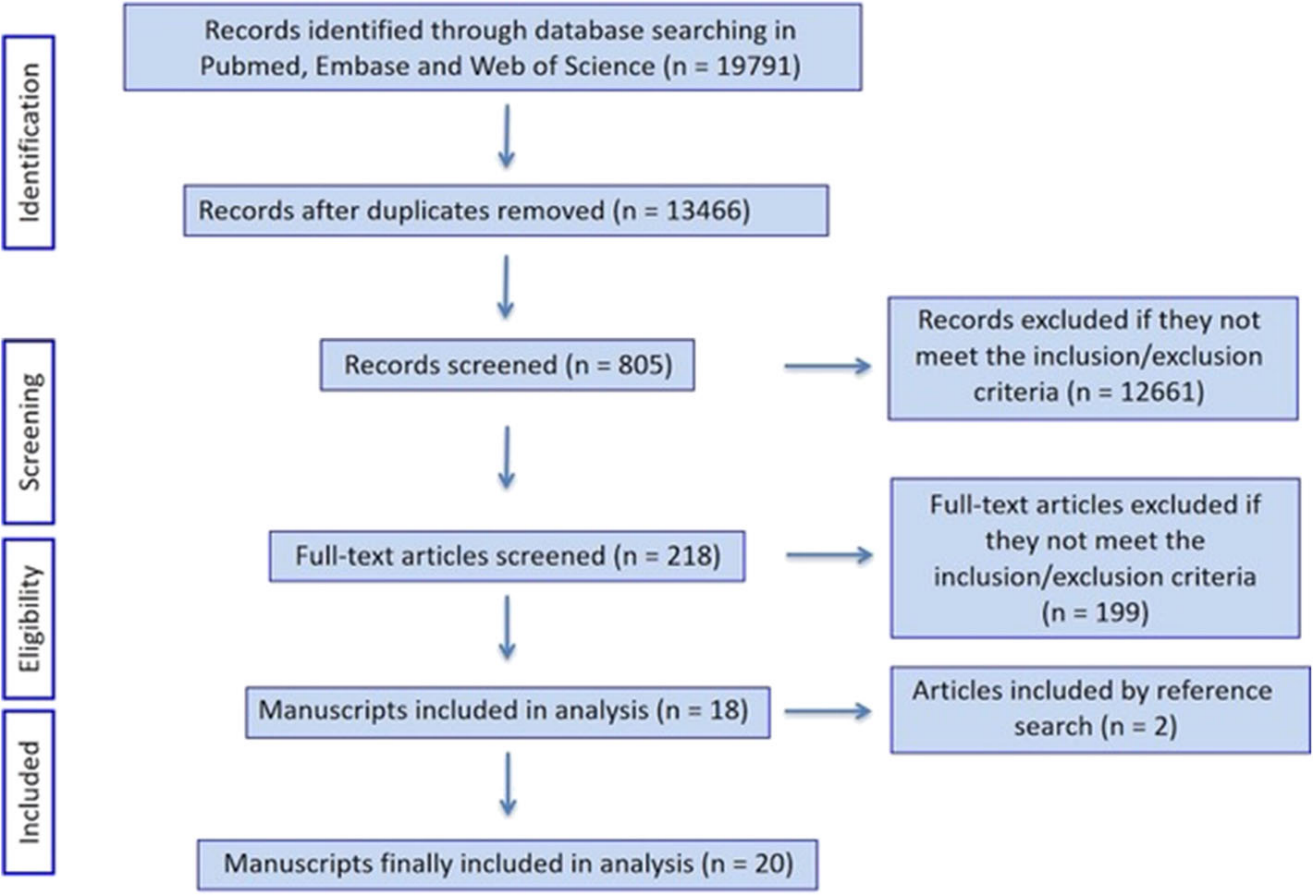
PRISMA flow diagram of the article search and screening for data extraction

The search returned 19,791 articles that were first screened and considered for inclusion in the review. Based on the inclusion criteria, the full texts of 218 articles were then reviewed, and 18 studies were retained. Two additional studies were included because they were cited by the included studies and considered relevant for this review. Twenty studies were therefore used in the final analyses (Fig. 1 and Table 1). The methodological quality of the assessed studies was considered moderate, with a mean score of 15 (55%), ranging between 12 (44%) and 18 (67%) (Table 2). Overall, data from 587 healthy individuals were analyzed: 227 children (4 studies), 310 young adults (16 studies), and 59 older adults (3 studies), in both treadmill (6 studies) and overground (14 studies) surfaces with a range of walking speeds. The mean ages of the participants per group were children 10.3 years, young adults 27.1 years, and older adults 69.2 years. For consistency, when available, gait speeds were reported in units of meters per second. However, two studies reported speeds only in dimensionless units [7, 30] and the speeds in meters per second were found in other studies that analyzed the same subjects [12, 56].

**Table 2:** Quality index assessment of the articles used in the final analysis

	Reporting (0–11)	External validity (0–3)	Internal validity—bias (0–7)	Internal validity—confounding (0–6)	Quality Index score (0–27) (%)
de David et al. 2015 [23]	8	1	5	0	14 (52)
Diop et al. 2005 [24]	9	1	5	2	17 (63)
Dubbeldam et al. 2010 [25]	8	1	5	1	15 (56)
Giarmatzis et al. 2015 [26]	9	1	5	2	17 (63)
Hsiao et al. 2015 [27]	6	1	5	0	12 (44)
Kerrigan et al. 1998 [28]	8	1	5	1	15 (56)
Khan et al. 2017 [29]	7	1	5	2	15 (56)
Kwon et al. 2015 [30]	6	1	4	1	12 (44)
Lewek 2011 [31]	8	1	5	1	15 (56)
Linden et al. 2002 [32]	5	1	5	1	12 (44)
Monaco et al. 2009 [33]	8	2	5	1	16 (59)
Ridge et al. 2016 [34]	9	1	5	2	17 (63)
Riley et al. 2001 [35]	7	1	5	0	13 (48)
Robbins et al. 2009 [36]	9	1	5	3	18 (67)
Schwartz et al. 2008 [7]	7	1	5	1	14 (52)
Silder et al. 2008 [37]	9	1	5	2	17 (63)
Wang et al. 2017 [38]	6	1	5	0	12 (44)
Weinhandl et al. 2017 [39]	8	1	5	2	16 (59)
Winiarski et al. 2019 [40]	9	3	5	1	18 (67)
Yang et al. 2013 [41]	7	1	5	1	14 (52)
				Mean	15 (55)

**Fig. 2:**
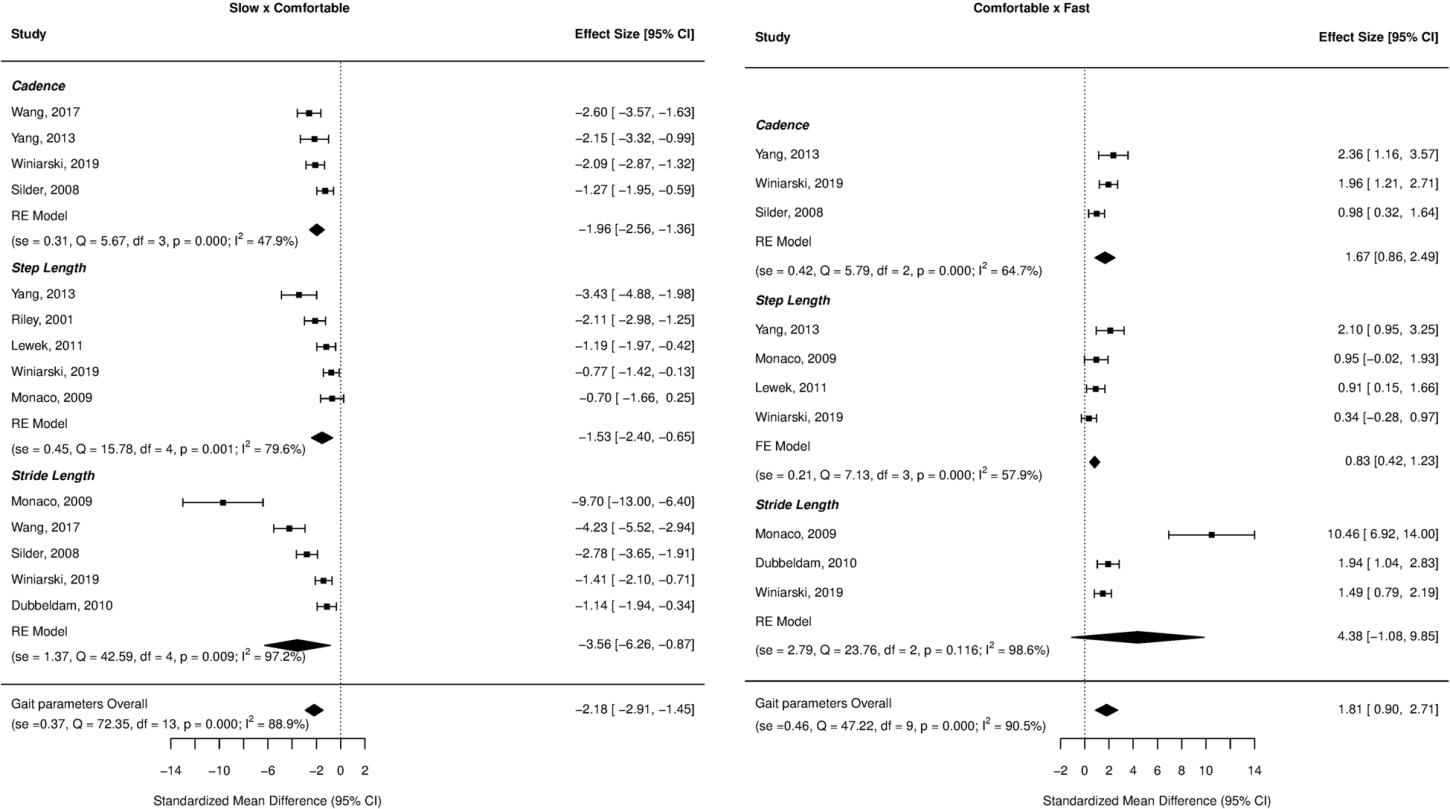
Forest plot of the gait parameters comparing the comfortable speed to the slow and fast speeds for the young adults

**Fig. 3:**
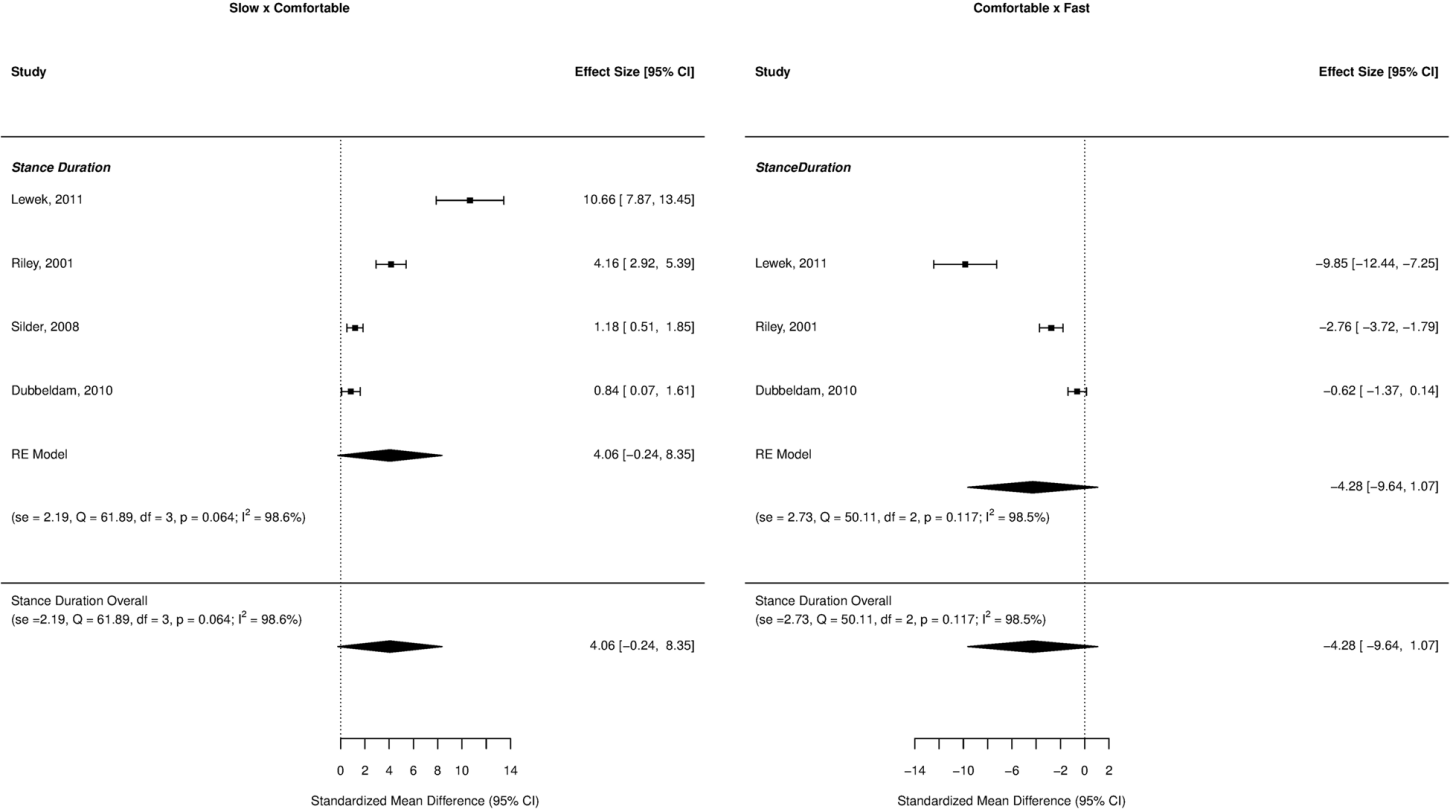
Forest plot of the stance duration comparing the comfortable speed to the slow and fast speeds for the young adults

**Fig. 4:**
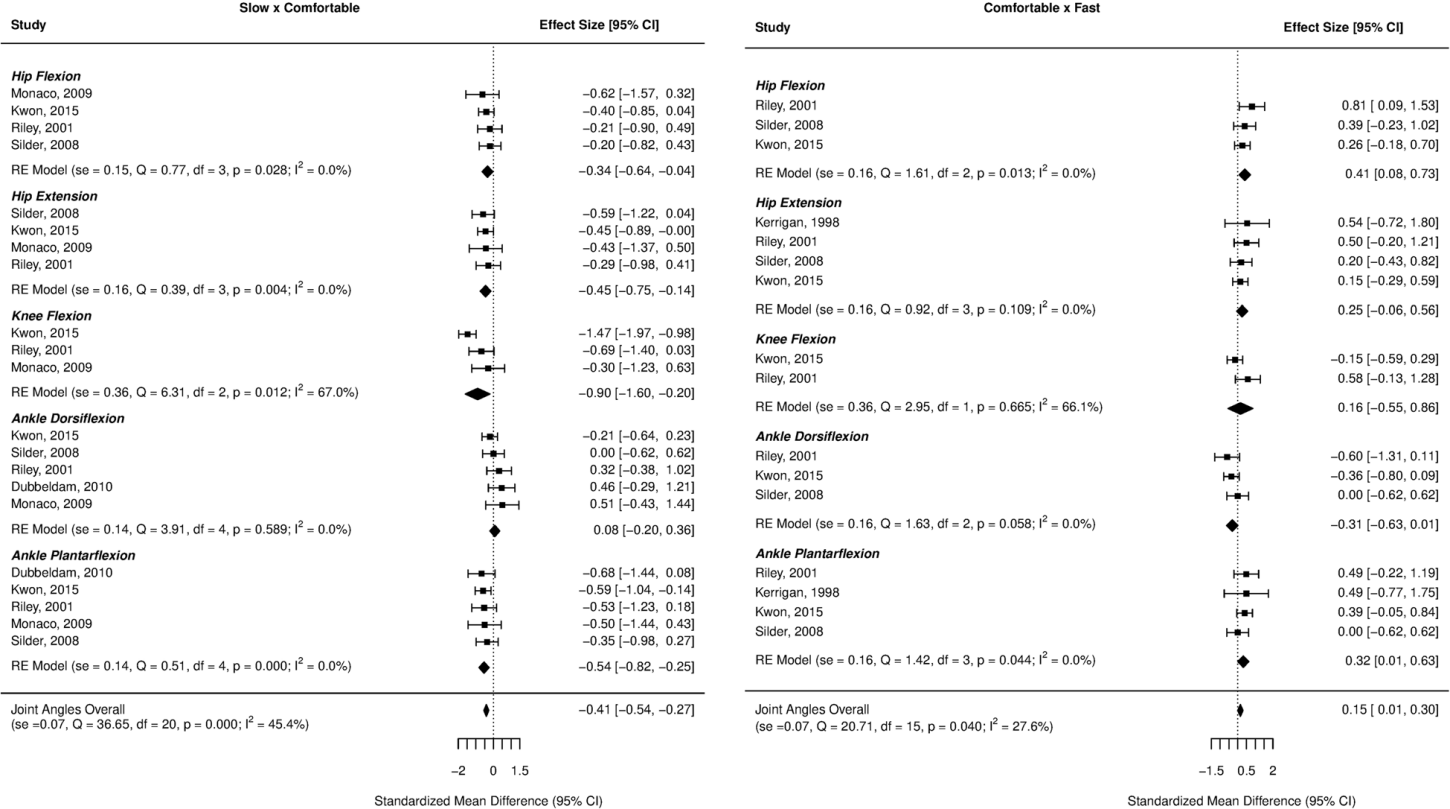
Forest plot of the joint angles comparing the comfortable speed to the slow and fast speeds for the young adults

**Fig. 5:**
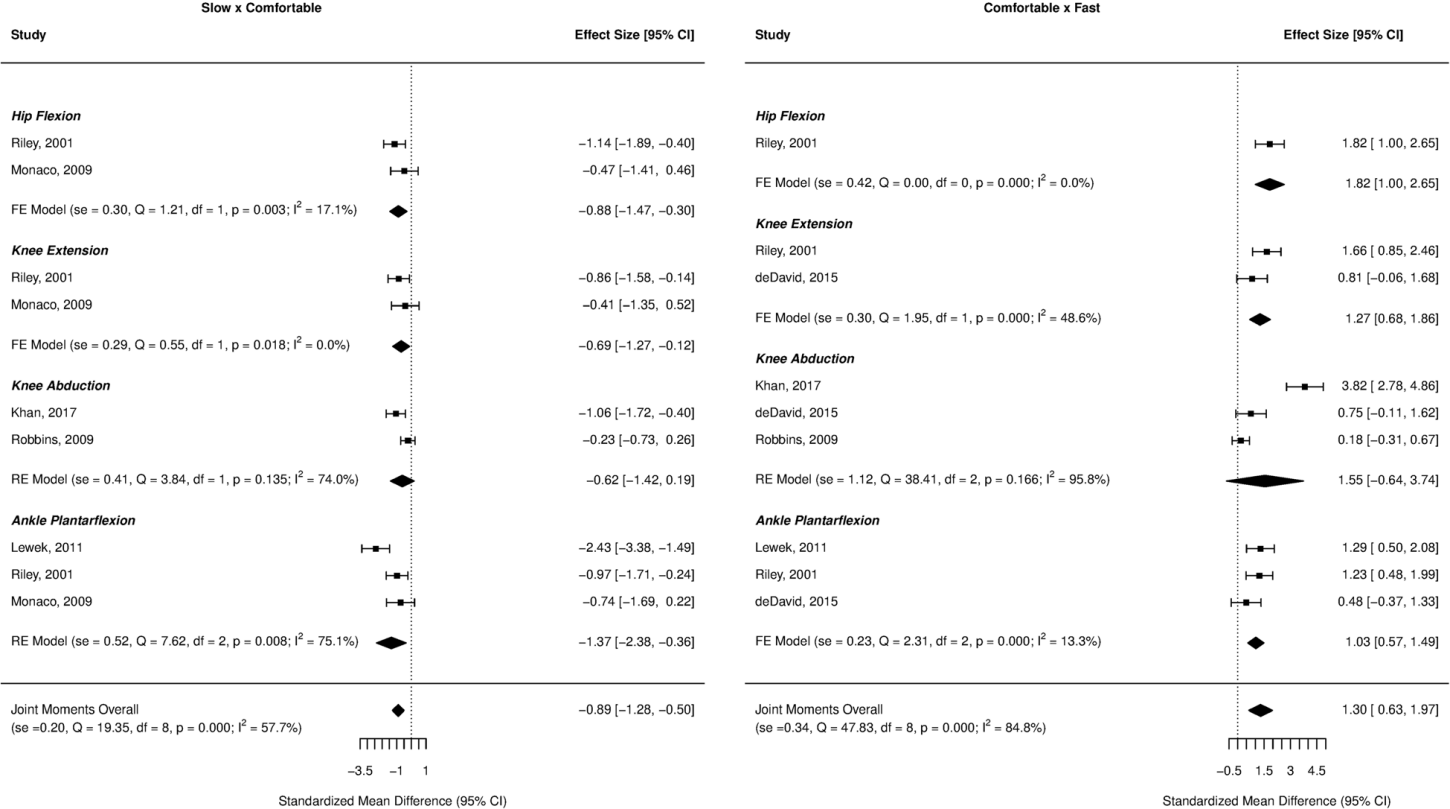
Forest plot of the joint moments comparing the comfortable speed to the slow and fast speeds for the young adults

**Fig. 6:**
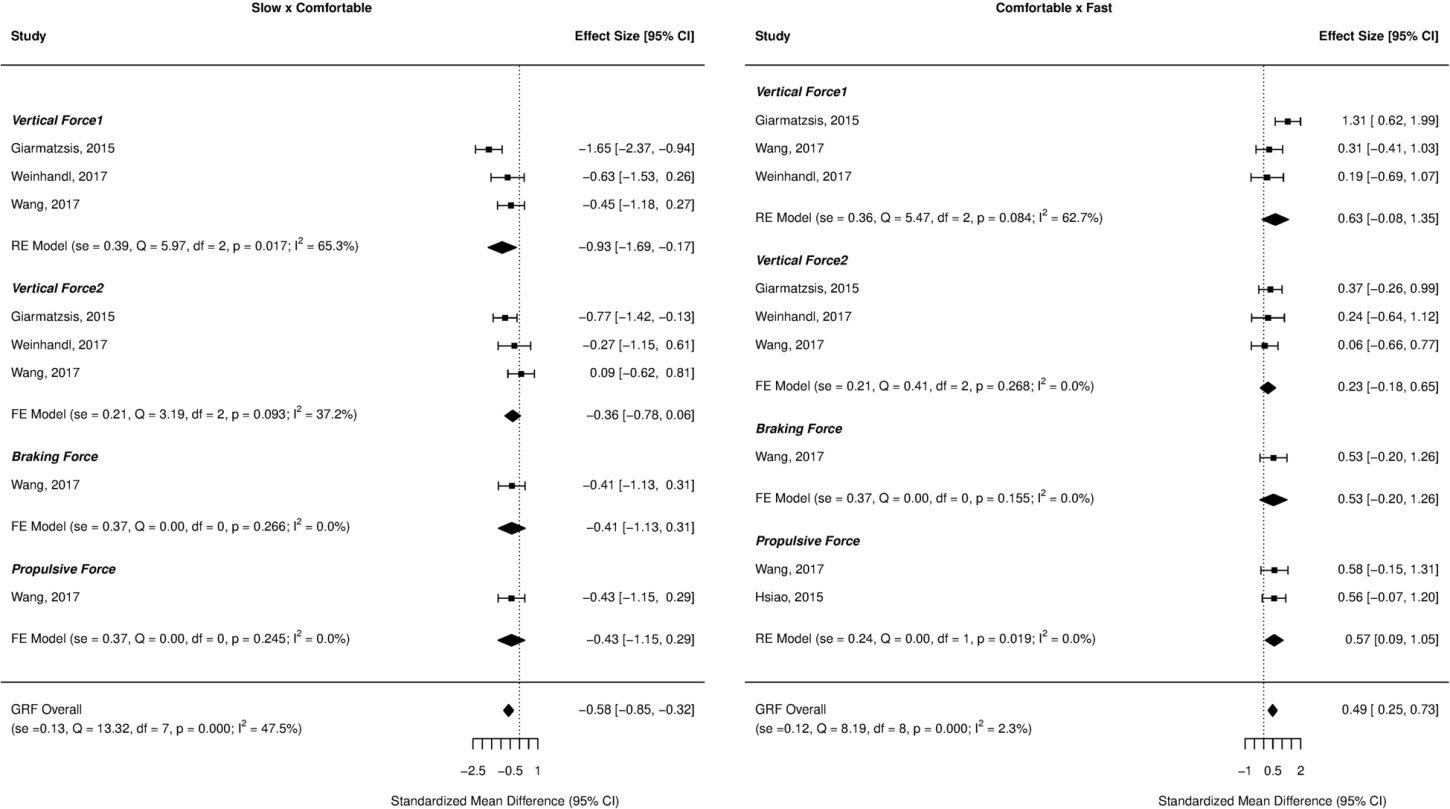
Forest plot of the ground reaction forces comparing the comfortable speed to the slow and fast speeds for the young adults

Forest plots for the investigated gait parameters are presented in Figs. 2, 3, 4, 5, and 6. Due to the small number of studies of children and older adults, their results were presented as a table instead of a forest plot in the supplemental material (Additional file 3: Table S2, Additional file 4: Table S3, Additional file 5: Table S4, and Additional file 6: Table S5). Specific changes in gait pattern due to walking speed were reported separately for each age group.

### Children

Gait speed influenced the spatiotemporal parameters in the child population. More specifically, large effects for cadence (ES = − 3.61, *p <* 0.001), step length (ES = − 3.29, *p <* 0.001), and stride length (ES = − 3.22, *p <* 0.001) were found during slower speeds, with a reduction in these variables when children walked slower. On the other hand, the stance duration (ES = 0.59, *p <* 0.001) presented a moderate effect, indicating an increase during slower speeds. At faster speeds, both cadence (ES = 2.97, *p <* 0.001) and step length (ES = 2.35, *p <* 0.001) presented large effect sizes, with higher values as the speed increased. Contrary to this, although there was also a large effect size for stance duration (ES = − 1.05, *p <* 0.001), its value decreased as the speed increased.

The joint kinematics showed large effect sizes for hip flexion (ES = − 0.80, *p <* 0.001), knee flexion (ES = − 1.34, *p <* 0.001), and ankle plantarflexion (ES = − 1.14, *p <* 0.001) angles, with decreases in their values as the speed decreased. There was a moderate effect for dorsiflexion angle (ES = 0.34, *p* = 0.031), but this increased at slower speeds. Regarding the fast speeds, a moderate effect was also found for ankle dorsiflexion angle (ES = − 0.63, *p <* 0.001), with a decrease in this at higher speeds.

For the joint kinetics, large effect sizes were found for the hip flexion (ES = − 1.70, *p <* 0.001) and knee extension (ES = − 1.52, *p <* 0.001) moments, and a moderate effect for the ankle plantarflexion moments (ES = − 0.60, *p <* 0.001). The results indicated that these variables decreased as walking speed decreased. In contrast, at faster speeds, the hip flexion, knee extension, and knee abduction moments increased as speed increased, with a moderate effect size for knee abduction (ES = 0.59, *p <* 0.001) and large effect sizes for hip flexion (ES = 1.84, *p <* 0.001) and knee extension (ES = 1.17, *p* = 0.024).

With regard to ground reaction forces, there were large effect sizes for the vertical1 (ES = − 1.21, *p <* 0.001), braking (ES = − 2.00, *p <* 0.001), and propulsive (ES = − 2.98, *p <* 0.001) forces, with lower values as the speed decreased. At faster speeds, these variables increased, with larger effect sizes for vertical1 (ES = 1.39, *p* < 0.001), braking (ES = 1.36, *p* < 0.001), and propulsive (ES = 1.50, *p* < 0.001) forces.

### Young adults

At slower speeds, the gait parameters showed large effect sizes for cadence (ES = − 1.96, *p* < 0.001), step length (ES = − 1.53, *p* = 0.001), and stride length (ES = − 3.56, *p* = 0.009), indicating a decrease when individuals walked slower. At faster speeds, there were large effect sizes for both cadence (ES = 1.67, *p* < 0.001) and step length (ES = 0.83, *p* < 0.001), indicating increases in these variables as the speed increased (Fig. 2).

For the joint kinematics at slow speeds, the effect sizes were small for the hip flexion (ES = − 0.34, *p* = 0.028) and extension angles (ES = − 0.45, *p* = 0.004), moderate for the ankle plantarflexion angle (ES = − 0.54, *p* < 0.001) and large for the knee flexion angle (ES = − 0.90, *p* = 0.012), indicating decreases in these variables as the speed decreased. Regarding the faster speeds, small effect sizes were found for the hip flexion (ES = 0.41, *p* = 0.013) and ankle plantarflexion (ES = 0.32, *p* = 0.044) angles, indicating an increase in these variables with faster speeds (Fig. 4).

The joint kinetics showed large effect sizes for the hip flexion (ES = − 0.88, *p* = 0.003) and ankle plantarflexion moments (ES = − 1.37, *p* = 0.008), and a moderate effect size for the knee extension moment (ES = − 0.69, *p* = 0.018), indicating that these values decreased as the speed decreased. In contrast, at faster speeds, there were large effects for the hip flexion (ES = 1.82, *p* < 0.001), knee extension moments (ES = 1.27, *p* < 0.001), and ankle plantarflexion moments (ES = 1.03, *p* < 0.001) indicating higher values at faster speeds (Fig. 5).

For the ground reaction forces, there was a large effect size for vertical1 (ES = − 0.93, *p* = 0.017), indicating a decrease at slower speeds. At faster speeds, the propulsive force showed a moderate effect size (ES = 0.57, *p* = 0.019), indicating an increase as the speed increased (Fig. 6).

### Older adults

For the older adult population, large effect sizes were found for the cadence (ES = − 1.86, *p* < 0.001), and step length (ES = − 1.14, *p* = 0.001) variables, indicating that both cadence and step length decreased when these individuals walked slower. When the individuals walked faster, there were large effect sizes for cadence (ES = 1.43, *p* < 0.001), step length (ES = 1.11, *p* = 0.001), and stride length (ES = 0.98, *p* < 0.001), indicating that these variables increased as the speed increased.

Regarding the joint angles and joint moments, significant effect sizes were found only at faster speeds. A moderate effect size was found for the hip flexion angle (ES = 0.57, *p* = 0.005), indicating an increase during faster speeds. For the joint moments, there were large effect sizes for both the hip flexion (ES = 1.01, *p* < 0.001) and knee extension (ES = 1.26, *p* < 0.001) moments, with these variables increasing as the speed increased.

## Discussion

The purpose of this systematic review and meta-analysis was to analyze the effects of walking speed on gait spatiotemporal parameters, joint kinematics, joint kinetics, and ground reaction forces in children, young adults, and older adults. We compared these variables during walking at either slow or fast speeds with walking at comfortable speeds. In total, 20 studies were included in this review; most of the variables were significantly affected by gait speed, with moderate-to-large effect sizes. Overall, the investigated variables presented smaller absolute amplitudes of the minimum and maximum values at slower speeds and larger absolute amplitudes at faster speeds. However, the effects of speed on gait biomechanics were not similar across the three analyzed groups.

The spatiotemporal gait parameters were generally affected by walking speed in all three age groups, with large effect sizes. Cadence and stride length have been reported as key determinants of walking speed in human locomotion [57]. The results found in this study are in agreement with previous studies where they reported a decrease in the duration of the stance phase with increased walking speed in children [10, 24]. Additionally, as speed increased, step length in both young adults and older adults, and stride length in older adults, also increased, corroborating the findings of a previous study [10].

In general, differences in joint kinematics, joint kinetics, and ground reaction forces due to changes in gait speed showed moderate-to-large effect sizes. Previous studies have reported the walking speed dependencies for these variables [6, 7, 11, 12, 50, 58, 59]. More specifically, for the child population, we observed that fast walking speeds were related to increased values in knee joint moments, in agreement with previous studies [7, 59]. In young adults, the effects of gait speed on the minimum and maximum values of joint angles have also been reported, including increases in hip flexion, hip extension, knee flexion, and ankle plantarflexion angles with higher speeds [50, 58, 60–62]. Applying a prediction method, a study by Lelas et al. [12] reported that even though most parameters changed with increasing gait speed, the predictability was better for the kinetic parameters compared to kinematics. For the older adults, the kinematic and kinetic variables were affected to a lesser extent than in either young adults or children because the differences were observed only at fast speeds, while the ground reaction forces did not change in any speed comparisons. Specifically, increases in the hip and knee flexion moments were found when older adults walked faster, which has also been reported in a previous study [61]. The fact that the observed changes only occurred at faster speeds in this age group might be explained by the aging effects which slows gait, and therefore the impact on slow walking would be smaller [63]. Additionally, when compared with the young adults walking at similar speeds, the older adults were less affected by the gait speed, presenting less knee extension at heel-strike and lower knee flexion during the swing phase [64]. Regarding the differences in the GRF, this variable was also affected by the gait speed but only in the children’s and young adults’ groups. Comparing these two groups, changes were more pronounced in the children’s group, where the vertical1, braking, and propulsive forces decreased at slower speeds and increased at faster speeds. This pattern at faster speeds is in agreement with a previous study [24]. In young adults, only the vertical1 force decreased at slow speeds, while the propulsive force increased at fast speeds, as per the findings of previous studies [65, 66].

Comparing the different age groups, while in the child population, the gait pattern has not matured yet and the speed seems to affect it to a greater extent [59], in older adults, as the rate of decline in walking speed is typically about 0.7% per year [67], the gait pattern suggests to be less affected by the speed. Therefore, the gait speed should also be considered when studying the effects of age in children and older adults. Moreover, as the minimum and maximum values of these specific biomechanical variables have been used to compare the gait patterns of pathological individuals who tend to walk slower than the control group [5, 68, 69], this comparison may be doable only after collecting data from a number of individuals walking at a variety of gait speeds, which is time-consuming and expensive. Rather, the use of public gait datasets [70–73] when available or the use of prediction methods are more appropriate alternatives to enable the establishment of reference gait patterns at different walking speeds [12, 58, 60, 62, 74, 75]. In fact, when a prediction method was applied to predict the gait pattern adjusting for a difference in gait speeds between groups, it has reduced the impact of gait speed on the calculation of gait indices such as the Gait Profile Score in post-stroke individuals [76].

This systematic review included the search of only three electronic databases (PubMed, Embase, and Web of Science) and this may be considered a limitation. However, these databases were selected for search because of their broad inclusion of multidisciplinary topics within the Biomedical and Health Sciences domain and because they have been particularly adopted in gait research reviews [18, 22, 64, 77]. In addition, only studies that employed 3D gait analysis instrumentation were included in this review and meta-analysis, which resulted in the majority of included studies being observational in nature. Therefore, while we acknowledge the risk of publication bias, it solely was likely not as important as the overall quality of studies which was assessed through a Quality Index tool [42].

## Conclusion

The results of this systematic review and meta-analysis show that speed affects the gait patterns of distinct age populations. Broader than previous reviews, where either only the knee moment or the spatiotemporal parameters was reported, this study analyzed the effects of speed on the gait pattern with respect to several gait parameters, including joint kinematics, kinetics, and ground reaction forces. In general, we observed that most of the absolute amplitude of the minimum and maximum values of the variables analyzed decreased at slower speeds and increased at faster speeds. The results of this study provide a stronger indication for the importance of also taking into account the effects of walking speed when comparing gait data of pathological individuals with normal or control individuals. Future studies involving such type of comparisons must control for the effects of different gait speeds, for example employing prediction methods in order to estimate the gait data of a normative group at the same speed of the pathological individual [75, 76].

## Additional files

Additional file 1: PRISMA 2009 Checklist. (PDF 89 kb)

Additional file 2: **Table S1.** Search strategy. (PDF 9 kb)

Additional file 3: **Table S2.** Meta-analysis for the comparison between slow × comfortable speeds for the children. (PDF 82 kb)

Additional file 4: **Table S3.** Meta-analysis for the comparison between comfortable × fast speeds for the children. (PDF 75 kb)

Additional file 5: **Table S4.** Meta-analysis for the comparison between slow × comfortable speeds for the older adults. (PDF 71 kb)

Additional file 6: **Table S5.** Meta-analysis for the comparison between comfortable × fast speeds for the older adults. (PDF 71 kb)

## Data Availability

The datasets used and/or analyzed during the current study are available from the corresponding author on reasonable request.

## Abbreviations

3D: Three-dimensional
CIs: Confidence intervals
ES: Effect size
GRF: Ground reaction forces
Q: Cochran’s heterogeneity statistic
QI: Quality Index
SEs: Standard errors
