# Supplementary material for: Effects of walking speed on gait biomechanics in healthy participants: a systematic review and meta-analysis
Source: Syst Rev. 2019 Jun 27;8:153. doi: 10.1186/s13643-019-1063-z (PMC6595586; doi:10.1186/s13643-019-1063-z)
Supplement: Supplementary file 2 — Table S1. Search strategy. (PDF 9 kb) [file 13643_2019_1063_MOESM2_ESM.pdf]

Table 1. Search strategy

1	Keywords	gait [or] walk*
2	Keywords	speed [or] velocit*
3	Keywords	kinematic* [or] kinetic* [or] biomech* [or] spatiotemporal [or] spatio-temporal [or] basic parameter$ [or] angle* [or] torque* [or] moment* [or] grf [or] ground reaction force*
4	Combine	1 [and] 2
5	Combine	4 [and] 5
