# Supplementary material for: Effects of walking speed on gait biomechanics in healthy participants: a systematic review and meta-analysis
Source: Syst Rev. 2019 Jun 27;8:153. doi: 10.1186/s13643-019-1063-z (PMC6595586; doi:10.1186/s13643-019-1063-z)
Supplement: Supplementary file 3 — Table S2. Meta-analysis for the comparison between slow × comfortable speeds for the children. (PDF 82 kb) [file 13643_2019_1063_MOESM3_ESM.pdf]

Table 2. Meta-analysis for the comparison between slow x comfortable speeds for the children.

Outcome Measures	# studies	ES	CI lower	CI upper	SE	Q	I^2	*p*-value
Gait parameters								
Cadence	2	-3.61	-4.02	-3.20	0.21	0.03	0.0%	<0.001*
Step Length	2	-3.29	-3.68	-2.90	0.20	0.54	0.0%	<0.001*
Stride Length	1	-3.22	-3.92	-2.52	0.36	0.00	0.0%	<0.001*
Stance Duration	1	0.59	0.29	0.88	0.15	0.00	0.0%	<0.001*
Joint angles								
Hip Flexion	1	-0.80	-1.11	-0.48	0.16	0.00	0.0%	<0.001*
Knee Flexion	1	-1.34	-1.68	-1.00	0.17	0.00	0.0%	<0.001*
Ankle Dorsiflexion	1	0.34	0.03	0.64	0.16	0.00	0.0%	0.031*
Ankle Plantarflexion	1	-1.14	-1.46	-0.81	0.17	0.00	0.0%	<0.001*
Joint moments								
Hip Flexion	1	-1.70	-2.06	-1.35	0.18	0.00	0.0%	<0.001*
Knee Extension	1	-1.52	-1.87	-1.18	0.18	0.00	0.0%	<0.001*
Knee Abduction	1	-0.16	-0.47	0.14	0.16	0.00	0.0%	0.289
Ankle Plantarflexion	1	-0.60	-0.91	-0.29	0.16	0.00	0.0%	<0.001*
Ground reaction forces								
Vertical1 Force	2	-1.21	-1.44	-0.99	0.12	0.00	0.0%	<0.001*
Vertical2 Force	2	-0.42	-0.98	0.13	0.28	6.88	85.5%	0.137
Braking Force	2	-2.00	-2.47	-1.52	0.24	3.37	70.3%	<0.001*
Propulsive Force	2	-2.98	-4.10	-1.86	0.57	13.08	92.4%	<0.001*
