# Supplementary material for: Effects of walking speed on gait biomechanics in healthy participants: a systematic review and meta-analysis
Source: Syst Rev. 2019 Jun 27;8:153. doi: 10.1186/s13643-019-1063-z (PMC6595586; doi:10.1186/s13643-019-1063-z)
Supplement: Supplementary file 4 — Table S3. Meta-analysis for the comparison between comfortable × fast speeds for the children. (PDF 75 kb) [file 13643_2019_1063_MOESM4_ESM.pdf]

Table 3. Meta-analysis for the comparison between comfortable x fast speeds for the children.

Outcome Measures	# studies	ES	CI lower	CI upper	SE	Q	I^2	*p*-value
Gait parameters								
Cadence	1	2.97	2.53	3.41	0.22	0.00	0.0%	<0.001*
Step Length	2	2.33	1.97	2.70	0.19	1.10	9.0%	<0.001*
Stance Duration	1	-1.05	-1.36	-0.75	0.16	0.00	0.0%	<0.001*
Joint angles								
Hip Flexion	2	0.60	-0.07	1.27	0.34	2.69	62.9%	0.079
Knee Flexion	2	0.90	-0.42	2.23	0.67	8.52	88.3%	0.180
Ankle Dorsiflexion	1	-0.63	-0.94	-0.32	0.16	0.00	0.0%	<0.001*
Ankle Plantarflexion	1	0.05	-0.26	0.35	0.16	0.00	0.0%	0.764
Joint moments								
Hip Flexion	2	1.84	0.92	2.76	0.47	3.35	70.1%	<0.001*
Knee Extension	2	1.17	0.15	2.19	0.52	4.91	79.6%	0.024*
Knee Abduction	1	0.59	0.28	0.90	0.16	0.00	0.0%	<0.001*
Ankle Plantarflexion	2	0.56	-0.19	1.31	0.38	3.32	69.9%	0.142
Ground reaction forces								
Vertical1 Force	2	1.39	1.15	1.62	0.12	0.01	0.0%	<0.001*
Vertical2 Force	2	0.36	-0.01	0.72	0.18	2.95	66.1%	0.054
Braking Force	2	1.36	0.99	1.73	0.19	2.58	61.3%	<0.001*
Propulsive Force	2	1.50	1.27	1.74	0.12	0.00	0.0%	<0.001*
