# Supplementary material for: Effects of walking speed on gait biomechanics in healthy participants: a systematic review and meta-analysis
Source: Syst Rev. 2019 Jun 27;8:153. doi: 10.1186/s13643-019-1063-z (PMC6595586; doi:10.1186/s13643-019-1063-z)
Supplement: Supplementary file 5 — Table S4. Meta-analysis for the comparison between slow × comfortable speeds for the older adults. (PDF 71 kb) [file 13643_2019_1063_MOESM5_ESM.pdf]

Table 4. Meta-analysis for the comparison between slow x comfortable speeds for the older adults.

Outcome Measures	# studies	ES	CI lower	CI upper	SE	Q	I^2	*p*-value
Gait parameters								
Cadence	2	-1.76	-2.84	-0.69	0.55	2.66	62.4%	0.001*
Step Length	1	-1.14	-1.81	-0.47	0.34	0.00	0.0%	0.001*
Stride Length	1	-1.06	-2.10	-0.01	0.53	0.00	0.0%	0.048*
Stance Duration	1	0.52	-0.48	1.52	0.51	0.00	0.0%	0.307
Joint angles								
Hip Flexion	2	-0.26	-0.79	0.26	0.27	0.16	0.0%	0.327
Hip Extension	2	-0.33	-0.86	0.20	0.27	0.14	0.0%	0.224
Knee Flexion	1	-0.66	-1.67	0.35	0.51	0.00	0.0%	0.198
Ankle Dorsiflexion	2	0.12	-0.41	0.64	0.27	0.21	0.0%	0.659
Ankle Plantarflexion	2	-0.10	-0.63	0.42	0.27	0.02	0.0%	0.704
Joint moments								
Hip Flexion	1	-0.52	-1.51	0.48	0.51	0.00	0.0%	0.310
Knee Extension	1	0.13	-0.85	1.11	0.50	0.00	0.0%	0.789
Ankle Plantarflexion	1	-0.86	-1.88	0.17	0.52	0.00	0.0%	0.100
