# Supplementary material for: Effects of walking speed on gait biomechanics in healthy participants: a systematic review and meta-analysis
Source: Syst Rev. 2019 Jun 27;8:153. doi: 10.1186/s13643-019-1063-z (PMC6595586; doi:10.1186/s13643-019-1063-z)
Supplement: Supplementary file 6 — Table S5. Meta-analysis for the comparison between comfortable × fast speeds for the older adults. (PDF 71 kb) [file 13643_2019_1063_MOESM6_ESM.pdf]

Table 5. Meta-analysis for the comparison between comfortable x fast speeds for the older adults.

**Outcome Measures**	**# studies**	**ES**	**CI lower**	**CI upper**	**SE**	**Q**	**I^2**	***p*-value**
Gait parameters								
Cadence	2	1.43	1.00	1.87	0.22	0.24	0.0%	<0.001*
Step Length	1	1.11	0.44	1.77	0.34	0.00	0.0%	0.001*
Stride Length	1	0.98	0.46	1.51	0.27	0.00	0.0%	<0.001*
Joint angles								
Hip Flexion	2	0.57	0.17	0.96	0.20	0.75	0.0%	0.005*
Hip Extension	2	0.11	-0.28	0.49	0.20	0.13	0.0%	0.594
Knee Flexion	1	0.47	-0.04	0.97	0.26	0.00	0.0%	0.070
Ankle Dorsiflexion	2	-0.29	-0.68	0.10	0.20	1.39	27.9%	0.148
Ankle Plantarflexion	2	0.12	-0.27	0.51	0.20	0.00	0.0%	0.548
Joint moments								
Hip Flexion	1	1.01	0.48	1.54	0.27	0.00	0.0%	<0.001*
Knee Extension	1	1.26	0.71	1.80	0.28	0.00	0.0%	<0.001*
Ankle Plantarflexion	1	-0.13	-0.63	0.37	0.25	0.00	0.0%	0.612
